# Potential Health Benefits of Fermented Vegetables with Additions of *Lacticaseibacillus rhamnosus* GG and Polyphenol Vitexin Based on Their Antioxidant Properties and Prohealth Profiles

**DOI:** 10.3390/foods13070982

**Published:** 2024-03-22

**Authors:** Chanya Ngamsamer, Chawanphat Muangnoi, Kullanart Tongkhao, Sudathip Sae-Tan, Khemmapas Treesuwan, Jintana Sirivarasai

**Affiliations:** 1 Doctoral Program in Nutrition, Faculty of Medicine Ramathibodi Hospital and Institute of Nutrition, Mahidol University, Bangkok 10400, Thailand; chanya.ngm@student.mahidol.edu; 2 Institute of Nutrition, Mahidol University, Nakhon Pathom 73170, Thailand; chawanphat.mua@mahidol.ac.th; 3 Department of Food Science and Technology, Faculty of Agro-Industry, Kasetsart University, Bangkok 10900, Thailand; kullanart.t@ku.ac.th (K.T.); fagists@ku.ac.th (S.S.-T.); 4 Institute of Food Research and Product Development, Kasetsart University, Bangkok 10900, Thailand; ifrkpt@ku.ac.th; 5 Nutrition Division, Faculty of Medicine Ramathibodi Hospital, Mahidol University, Bangkok 10400, Thailand

**Keywords:** fermented vegetables, *Lacticaseibacillus rhamnosus* GG, polyphenol vitexin, microbiota, antioxidant activity

## Abstract

Fermented vegetables are increasingly being recognized as an important dietary component, particularly of plant-based diets, to achieve a sustainable healthy gut because of their microbial diversity and antioxidant properties. However, the functional relevance of fermented vegetables varies based on the raw ingredients used and nutrient supplementation. Therefore, in the present study, we investigated the microbial diversity and antioxidant activity of three formulas of fermented vegetables (standard, supplemented with *Lacticaseibacillus rhamnosus* GG, and supplemented with polyphenol vitexin) at days 0 and 15. The bacterial community profiles were determined through 16S rRNA sequencing analysis, and antioxidant activity was analyzed using 2,2-diphenyl-1-picrylhydrazyl and by measuring the oxygen radical absorbance capacity, the ferric reducing ability of plasma, and the total phenolic content. The results confirm microbial diversity in the taxonomic composition of the different formulas of fermented vegetables, with different bacteria predominating, particularly lactic acid bacteria including the genera *Weissella*, *Pedicocccus*, *Leuconostoc*, and *Lactobacillus*. Spearman’s correlation analysis showed significant differences in the specific bacteria present in the different formulas of fermented vegetables that conferred antioxidant capacity. Our findings show that supplementation with *L. rhamnosus* GG and polyphenol vitexin may effectively enhance the functional relevance of foods by promoting cellular protection against oxidative stress.

## 1. Introduction

The current obesity pandemic constitutes an ongoing global public health burden by contributing to health conditions such as diabetes, cardiovascular disease, nonalcoholic fatty liver disease, and obesity-associated cancers [1]. Obesity and its comorbidities involve complex mechanisms and multifactorial interactions; therefore, the challenge is to reduce potential underling disease pathways, such as oxidative stress, inflammation, and the dysbiosis of the gut microbiota [2]. A variety of diets have been promoted as therapeutic strategies for obesity, with a plant-based diet being one of the main recommendations for dietary management [3].

Many plant-based fermented foods, including kimchi, natto, sauerkraut, tofu, miso, and sourdough bread, are consumed extensively throughout the world. Such foods provide the macro- and micro-nutrients required for beneficial health outcomes as a result of their various functional properties such as their hypolipidemic effects, their anticancer, antihypertensive, antidiabetic, antioxidant, and immune-promoting capacities [4]. Additionally, all fermented vegetables have probiotic properties. Metabolites of lactic acid bacteria (LAB) possess biologically active compounds, biogenic amines, and biologically active compounds from phenolic compounds [4]. The genera of LAB present in fermented foods include *Lactobacillus*, *Lactococcus*, *Pediococcus*, *Enterococcus*, *Streptococcus*, *Leuconostoc*, and *Weissella*, among others [5]. The microbial community present within fermented vegetables is affected by the composition of ingredients, the nutritive components, the duration of fermentation, and the physical conditions [5]. In addition, the LAB present in fermented vegetables determine the quality and safety of the fermented vegetables and confer potential health benefits as a result of their anti-inflammatory, immunomodulatory, and gut health-promoting properties [6].

In the present study, we focused on the potential health benefits of fermented vegetables, fermented through a process similar to that of kimchi, a traditional Korean food. Previous studies showed that *Lactococcus lactis* KC24 and *Lactobacillus plantarum* KCCM 11352P isolated from kimchi exhibit antioxidant, anti-inflammatory, and anticancer effects, and are therefore potential novel probiotics in functional foods [7,8]. Yun et al. found that commercial fermented vegetables in Korea had higher antioxidant activities than those in China and the USA because of differences in the physiological properties of the ingredients and the higher number of operational taxonomic units (OTUs), indicating a wider variety of microorganisms [9]. Lee et al., reported a significantly higher radical scavenging activity, reducing power, and lipid peroxidation inhibition in LAB strains isolated from kimchi, specifically *Latilactobacillus curvatus*, *Companilactobacillus allii*, and *L. lactis*, than in other strains [10]. In addition, based on transcriptomic and proteomic data, a notable molecular function of these three LAB strains was their response to oxidative damage that was related to changes in the cell membrane, intracellular metabolism, and cellular processes [10].

Considering the various functional benefits of LAB present in fermented foods, the production of probiotic fermented foods is a significant trend in the worldwide fermented foods business [11]. This study selected *Lacticaseibacillus rhamnosus* GG probiotic strains grown in fermented milk to test a model for probiotic fermented food production. The results indicate that only an initial inoculum of 106 colony-forming units (CFU)/mL fulfilled the requirements of probiotic dairy products, supporting survival and growth under the thermal and nutritional conditions typical of dairy cultures [11]. However, many published reports have stated the importance of *L. rhamnosus* in dairy products and beverages. The viability of the probiotic *L. rhamnosus* GG strain and the physicochemical properties of semisolid kurut products (a traditional acid-coagulated fermented dairy product) during storage over 22 days at 4 °C have been reported, indicating their high potential as probiotic products to be produced in the dairy industry [12]. In another report, a DPPH assay revealed the antioxidant activity of fermented tomato juice during refrigerated storage and the viability (>7 log CFU/mL) of *L. rhamnosus* (ATCC 53103) and *Lacticaseibacillus casei* (ATCC 393) after a 120 min exposure to simulated gastric and intestinal fluids [13]. In addition, the remarkable oxidative stress resistance of *L. rhamnosus* (AS 1.2466) has been evidenced with the coexpression of the superoxide dismutase and catalase, potentially indicating a valuable probiotic starter culture in industrial fermentation [14]. Currently, available data for *L. rhamnosus*-supplemented fermented vegetables are limited. To address this gap in knowledge and simultaneously facilitate the production of fermented vegetables, we selected an *L. rhamnosus* strain for the supplementation of standard formular with the potential health-promoting attributes of certified probiotic strains.

Furthermore, a number of studies have proposed the potential functional activities of polyphenols in plant-fermented foods and suggested that these foods have higher bioavailability and bioactivity than foods rich in polyphenols, whilst also promoting the growth of probiotic bacteria and conferring potent antioxidant properties [15,16]. The potential of polyphenols as fermentable substrates in functional foods leads to the possibility of developing novel functional foods. Vitexin (apigenin-8-C-β-D-glucopyranoside) is a polyphenol, belonging to the class of flavones, that is found as a major polyphenol in food sources such as mung beans. Vitexin plays a major role as an antioxidant and functions by decreasing the levels of reactive oxygen species, inhibiting lipid peroxidation, decreasing the expression of NOX4, and inhibiting the release of cytochrome c from mitochondria into the cytoplasm [17]. Previous studies showed that the antioxidant capacities of vitexin and isovitexin in mung bean soup accounted for most of the radical scavenging (as determined using DPPH and ABTS) and ferric reducing activities [18,19].

To our knowledge, this is the first study to investigate the association between three groups of fermented vegetables (i.e., standard fermented vegetables, *L. rhamnosus* GG-supplemented fermented vegetables, and polyphenol vitexin-supplemented fermented vegetables) with antioxidant and probiotic properties on potential health impacts. In addition, another outcome of this study involved providing evidence of design for new product development.

## 2. Materials and Methods

### 2.1. Materials

Fermented vegetables were prepared using Chinese cabbage, garlic, ginger, onion, carrot, radish, and spring onion (all supplied by Makro, Bangkok, Thailand); low-sodium salt and low-sodium fish sauce (Good Life, Nakornpathom, Thailand); red pepper powder (Gochugaru, Bangkok, Thailand); glutinous rice flour (Erawan brand, Nakornpathom, Thailand), low-calorie sugar (Sucrose, Mitr Phol, Suphanburi, Thailand); and water (Nestle, Phra Nakhon Si Ayutthaya, Thailand). The commercial probiotic culture used in this study was *Lacticaseibacillus rhamnosus* GG (Chr. Hansen, Honsholm, Denmark). Mung bean seed coat (MBC), a co-product of the mung bean dehulling process, was received from Kittitat Co., Ltd. (Bang Khun Thian, Bangkok, Thailand).

Folin–Ciocalteu reagent, saturated sodium carbonate solution (Na_2_CO_3_), gallic acid, 1,1-diphenyl-2-picrylhydrazyl radical (DPPH), ethanol, Trolox, potassium di-hydrogen phosphate (KH_2_PO_4_), di-potassium hydrogen phosphate (K_2_HPO_4_), fluorescein sodium salt, 2′-azobis (2-amidinopropane) di-hydrochloride (AAPH), sodium acetate trihydrate, 2,4,6-tripyridly s-triaine (TPTZ), acetate buffer, ferric chloride hexahydrate (FeCl_3_·6H_2_O), and HCl (Sigma-Aldrich, St. Louis, MO, USA) were used for the total phenolic content (TPC), DPPH-radical scavenging, oxygen radical absorbance capacity (ORAC), and ferric-reducing antioxidant power (FRAP) assays. De Man, Rogosa, and Sharpe agar (MRS; Merck, Darmstadt, Germany) and vancomycin (MRS-50V) (Sigma-Aldrich) were used for total bacterial counts.

### 2.2. Production of Fermented Vegetables

The fermentation process in our study involved the selection of fresh vegetables, washing and removing any rotten and moldy parts, cutting the vegetables into the desired shapes to facilitate the pickling process, and placing them in a container. The Chinese cabbage (55–90% *w*/*w*) was thoroughly washed, salt-cured with low-sodium salt (1–10% *w*/*w*), and then left for approximately 5 h at room temperature. Seasonings were thoroughly mixed with garlic (1–5% *w*/*w*), ginger (0.1–5% *w*/*w*), and onion (1–10% *w*/*w*), and then low-sodium fish sauce (1–9% *w*/*w*) and red pepper powder (0.1–7% *w*/*w*) were added. Next, glutinous rice flour (1–5% *w*/*w*) and water (1–25% *w*/*w*) with low-calorie sugar (55–90% *w*/*w*) were added, and then chopped carrot (0.1–10% *w*/*w*), radish (0.1–10% *w*/*w*), and spring onion (0.1–10% *w*/*w*) were mixed in. Subsequently, the vegetables were rinsed, and then the seasoning was spread over the vegetables. The fermentation of the vegetables was carried out in sealed containers for 36 h at room temperature.

#### 2.2.1. Preparation of Probiotic-Fermented Vegetables

Lyophilized *Lactobacillus rhamnosus* LGG^®^ (LGG) was purchased from Chr. Hansen A/S (Hoersholm, Denmark). During the preparation of probiotic-fermented vegetables, 24 h fermented vegetables were divided into two parts: fermented broth and fermented vegetables. Then, 0.01 g of LGG was inoculated into 5 mg of fermented broth and mixed well. Next, the LGG inoculated broth was added to 45 g of fermented vegetables to reach a final concentration at 108 CFU/g, and this mixture was stored at 4 °C for 15 days. The LGG inoculated probiotic-fermented vegetables were sampled on days 0, 3, 6, 9, 12, and 15 for microbial and acidity analyses.

#### 2.2.2. Preparation of Vitexin-Fermented Vegetables

Mung bean seed coat (MBC) was purchased from Kittitat Co., Ltd. (Thailand). The raw material was kept at 0 °C before use. For the preparation of MBC extract, polyphenols were extracted from MBC with 50% ethanol using pressurized liquid extraction [20]. The conditions for extraction were 1300 PSI at 160 °C. The extract was filtered and concentrated using a rotary evaporator, and then centrifuged at 7000 rpm for 10 min at 25 °C. The concentrated extract was dried with a pilot spray dryer using maltodextrin and gum arabic as carriers. The obtained polyphenol extract was stored at −20 °C in an aluminum bag before use.

For the preparation of another fermented vegetable formula, polyphenol extract was dissolved in the liquid from the fermented vegetables, and after thorough mixing, the solution was added back to the fermented vegetables. The fermented vegetables supplemented with polyphenol extract were stored at 4 °C before analysis.

**Figure 1:**
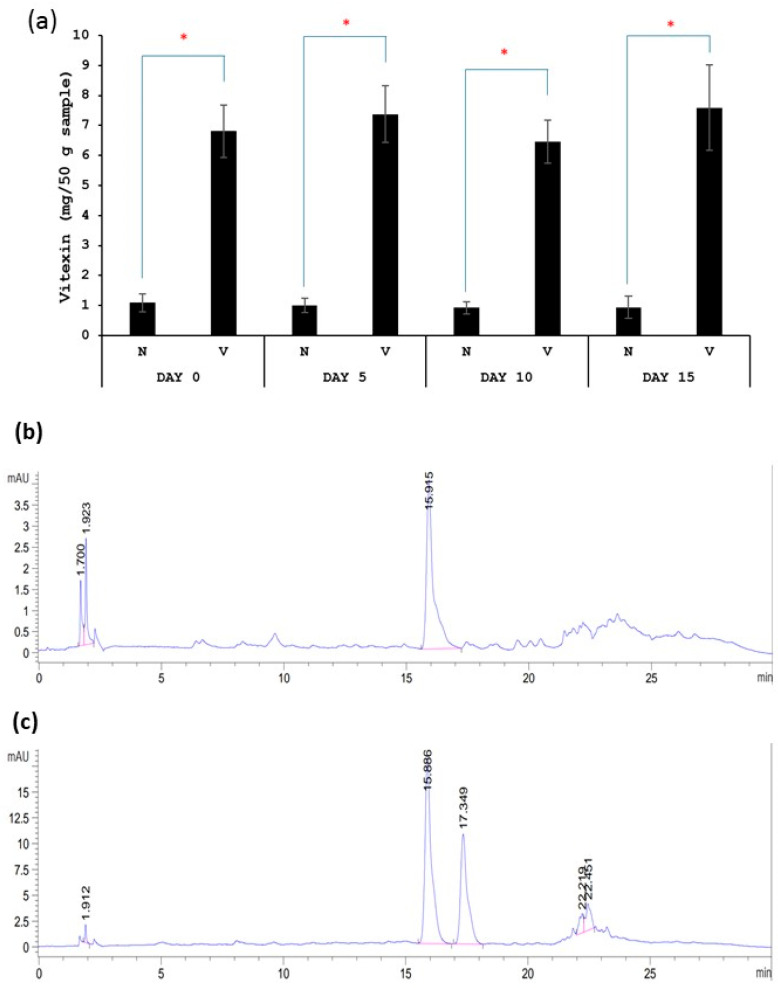
Vitexin content of standard fermented vegetables (N sample) and fermented vegetables with vitexin (V sample) (**a**) and chromatographic analysis of standard fermented vegetables (**b**) and fermented vegetables with vitexin (**c**). Retention time of vitexin is at 15.8 min. * indicates the statistically significant difference between N sample and V sample.

Vitexin quantification was conducted as described below. First, polyphenol was extracted from kimchi samples according to the previously reported method by Kim, Lee et al. (2018) [21]. Briefly, kimchi was ground with a blender. Then, 5 g of ground kimchi was mixed with 25 mL of 60% acetonitrile and vortexed for 1 min. The mixture was sonicated at 37 °C for 10 min. Then, the mixture was centrifuged at 3000 rpm for 10 min. The supernatant was collected and filtered through a syringe filter (0.45 µM) before the determination of the vitexin content using HPLC according to Pavasutti, Sinthuvanich et al. (2023) [22]. Samples were subjected to HPLC equipped with a diode array detector (DAD) (Waters 600, Spectralab Scientific Inc., Milford, MA, USA). The absorption spectra were recorded from 210–600 nm for all peaks. UV absorbance at 280 nm and 337 nm was used to monitor phenolic compounds and flavonoids, respectively. Samples were passed through an analytical column C18 (4.6 × 250 mm, 5 µm, Waters Symmetry Column, Agilent Technologies Inc., Dublin, Ireland) and were stored at 30 °C. The injection volume of the sample was 10 µL. Elution was performed using two solvent gradients: solvent A (1% acetic acid in deionized water, *v*/*v*) and solvent B (1% acetic acid in methanol, *v*/*v*). Elution was carried out at a flow rate of 1 mL/min. The gradient program was as follows: 10–35% B (10 min), 35–42% B (15 min), 42–75% B (10 min), 75% B (5 min), 10–75% B (5 min), and 10% B (5 min). We obtained the preliminary results of the vitexin content and chromatographic analysis of the standard formula (N) and fermented vegetables with vitexin (V), shown as follows. Figure 1a shows that the vitexin content in fermented vegetables with vitexin (V sample) was significantly higher than that in standard fermented vegetables (N sample). The vitexin contents of the standard fermented vegetables and fermented vegetables with vitexin were stable during a 15-day storage time. Figure 1b,c show chromatograms of the standard fermented vegetables and fermented vegetables with vitexin, respectively. The chromatograms show the peak of vitexin at the retention time of 15.8 min, which agrees with the standard vitexin. The second peak in Figure 1c is isovitexin. This agrees with a previous study that extracted polyphenols from mung bean seed coat extract [20], and the present study used the same method.

All fermented vegetable samples were stored for 15 days at 4 °C and sampled on days 0, 3, 6, 9, 12, and 15. The fermented vegetables samples were analyzed according to quality indexes (pH, LAB, and *L. rhamnosus* GG count). Moreover, sampling at 4 °C from days 0 to 15 was performed to investigate the microbial diversity and antioxidant activity.

### 2.3. pH Determination

The fermented vegetables were periodically sampled at 0, 3, 6, 9, 12, and 15 days, and the pH values of the samples were measured using a pH meter (OAKTON PH 2700, Ayer Rajah Crescent, Singapore).

### 2.4. Determination of the Antioxidant Activity of Fermented Vegetables

First, samples were freeze-dried (LD-0.5, Kinetic Engineering Co., Ltd., Bangkok, Thailand). Then, 1 g of powder fermented vegetable samples was extracted with distilled water (15 mL) according to the method described by Park et al. [23] with a slight modification. The aqueous solution was shaken at room temperature for 12 h. Then, following filtration through Whatman filter paper, the TPC, DPPH radical scavenging activity, ORAC, and FRAP were measured.

#### 2.4.1. Total Phenolic Content (TPC)

The TPC was measured using the Folin–Ciocalteau method [24]. Briefly, 160 μL of distilled water, 10 μL of sample extract, 20 μL of saturated sodium carbonate solution (75% *w*/*v*), and 10 μL of Folin–Ciocalteu reagent were added to a 96-well plate. After incubation for 90 min at room temperature, the absorbance was measured at 750 nm. The TPC was determined using a standard curve for gallic acid and was expressed as µg of gallic acid equivalent (GAE) per g of sample.

#### 2.4.2. DPPH Free Radical Scavenging Activity

The DPPH free radical scavenging activity was measured as described by Amarowicz et al. [25]. Briefly, 20 μL of sample extract and 100 μL of DPPH reagent (150 μM) were added to a 96-well plate. After incubation for 30 min at room temperature, the absorbance was measured at 520 nm. The DPPH was determined using a standard curve for Trolox.

#### 2.4.3. Oxygen Radical Absorbance Capacity (ORAC)

ORAC was measured according to the method described by Huang et al. [26]. ORAC working buffer solution was prepared by first generating an ORAC buffer stock solution comprising 603 mL potassium di-hydrogen phosphate (0.75 M) mixed with 351 mL dipotassium hydrogen phosphate (0.75 M), and then diluting 100 mL of this stock solution with 900 mL of distilled water. Additionally, fluorescein working solution was prepared by first generating a fluorescein concentrate solution (8.37 × 10^−4^ mM) comprising 0.045 g fluorescein sodium salt, adjusted to 100 mL with ORAC working buffer solution, and then diluting 50 μL of this concentrate solution with 9950 μL ORAC working buffer solution, and then finally diluting 1.95 mL fluorescein stock solution with 98.05 mL ORAC working buffer solution.

Briefly, 25 μL of sample extract, 25 μL of standard Trolox solution, 25 μL of blank (ORAC working buffer solution), and 150 μL of fluorescein working solution were added to a 96-well plate. After incubating for 30 min at 37 °C, 25 μL of 2′-azobis (2-amidinopropane) di-hydrochloride (AAPH) was also added to the 96-well plate. The absorbance was measured at an excitation wavelength of 485 nm and an emission wavelength of 540 nm every 1 min for up to 150 min using a fluorescein microplate reader. ORAC values were determined using a standard curve for Trolox and were expressed as µmol of Trolox equivalent per g of sample.

#### 2.4.4. Ferric Reducing Antioxidant Power (FRAP)

The FRAP values of samples were measured as described by Halvorsen et al. [27]. First, FRAP reagent solution was prepared with 10 mL of acetate buffer (300 mM) and adjusted to 1000 mL by adding distilled water. Next, 1 mL of 2,4,6-Tris(2-pyridyl)-s-triazine (10 mM) dissolved in HCl (40 mM) and 1 mL of ferric chloride hexahydrate (20 mM) dissolved in distilled water were mixed. Then, 20 μL of sample extract and 150 μL of FRAP reagent were added to a 96-well plate. Then, the plate was incubated for 8 min at room temperature. FRAP was measured at 600 nm with a standard curve for Trolox.

### 2.5. Microbial Counts

The total number of LAB (natural lactic microflora + probiotic) in the samples was quantified on de Man, Rogosa, and Sharpe (MRS; Merck, Darmstadt, Germany) agar with 50 ppm vancomycin (MRS-50V) and anaerobically incubated at 37 °C for 2 days, as recommended by the Chr. Hansen Company. The vancomycin was purchased from Sigma-Aldrich. Total LAB were quantified on MRS agar with no vancomycin added, and incubated under anaerobic conditions at 37 °C for 2 days. The pH values were measured using a pH meter (Oakton model pH2700, Ayer Rajah Crescent, Singapore). All experiments were performed in triplicate.

### 2.6. DNA Extraction and Sequencing

Microbial DNA was extracted from all fermented vegetables using the DNeasy PowerSoil Pro kit (Qiagen, Germantown, MD, USA). Nanodrop spectrophotometry and electrophoresis were used to evaluate the quantity and quality of the DNA. The V4 hypervariable region of the 16S rRNA gene was amplified via PCR using the 515 F and 806R primers and 2× KAPA hot-start ready mix. The PCR conditions included an initial denaturation at 94 °C for 3 min, followed by 25 cycles of 98 °C for 20 s, 55 °C for 30 s, 72 °C for 30 s, and a final extension step at 72 °C for 5 min. The 16S amplicons were purified using AMPure XP beads and indexed using the Nextera XT index kit, followed by 8 cycles of the aforementioned PCR conditions. The PCR products were then cleaned and pooled in preparation for cluster generation and Illumina^®^ MiSeq™ 250-bp paired-end read sequencing.

### 2.7. Microbiome Bioinformatic Analysis

Microbiome bioinformatics was performed with QIIME 2 2022.2 [28]. The raw sequence data were demultiplexed using the q2-demux plugin, and reads with expected errors (maxEE) higher than 3.0 were discarded by employing denoising software, DADA2 (via q2-dada2 plugin QIIME2 Version 2022.2). A total of 16S sequences belonging to chloroplasts and mitochondria were also removed. A phylogeny was constructed from the representative sequences using the q2-phylogeny plugin align_to_tree_mafft_fasttree action. Beta-diversity metrics were estimated using q2-diversity after the samples were rarefied (subsampled without replacement) to reads, and principle coordinate analysis (PCoA) was conducted. Taxonomy was assigned to amplicon sequencing variants using the classify-sklearn naive Bayes taxonomy classifier against the SILVA reference sequences. A statistical analysis of the beta diversity was performed using PERMANNOVA (number of permutations = 999), and a differential abundance analysis of microbiota was conducted using nonparametric factorial Kruskal–Wallis sum-rank tests, followed by a Wilcoxon rank sum test correcting for multiple comparisons.

### 2.8. Statistical Analysis

Statistical analyses were performed using IBM SPSS software version 22. The results are presented as the mean ± standard deviation (SD). Statistical significance was analyzed using a paired t-test and a one-way analysis of variance (ANOVA), followed by a Tukey HSD test. *p* values < 0.05 were considered statistically significant.

## 3. Results

### 3.1. Changes in the pH Values of Fermented Vegetables

**Figure 2:**
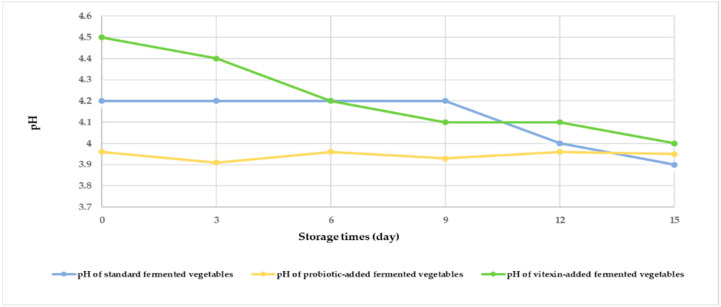
Measurement of the pH of each of the fermented vegetable formulas during the storage period (days 0, 3, 6, 9, 12, and 15).

Changes in the pH values of the three fermented vegetable formulas during storage are shown in Figure 2. At the beginning of fermentation, the pH of the three fermented vegetable formulas ranged from 3.90 to 4.50. The pH of the standard fermented vegetables did not change during storage from days 0–9 but decreased on days 12 and 15, while the pH of the probiotic-fermented vegetables also decreased on days 3 and 9. The vitexin-fermented vegetables showed a gradual decrease in pH during the storage period.

### 3.2. Microbiological Analyses

Besides the enumeration method using MRS-50V for *L. rhamnosus* LGG^®^ (LGG) recommended by the Chr. Hansen company some colonies on the MRS-50V plate were sampled to reaffirm its strain as LGG using MALDI-TOF MS and DNA sequencing techniques. These protocols were used as internal validation protocols for our laboratory work. The changes in the microbial counts of the probiotic-fermented vegetables during storage were determined and are presented in Table 1. During the 15-day fermentation stage, *L. rhamnosus* GG counts ranged from 7.71 to 7.48 log CFU/g. There were no significant differences in the *L. rhamnosus* GG counts during the storage of fermented vegetables at 4 °C for 15 days. For LAB, the initial viable count at 4 °C was 8.53 log CFU/g, and this remained stable from days 3 to 9, whereas on days 12 and 15, there was a significant decrease in viable count compared with that at the earlier time points. These results indicate that probiotic-fermented vegetables contain viable bacteria (≥106 CFU/g) throughout the shelf-life of this product at 4 °C.

### 3.3. Antioxidant Activity and Total Phenolic Content

The total phenolic content and three parameters of antioxidant activity for the three fermented vegetable formulas (standard fermented vegetables, probiotic-fermented vegetables, and vitexin-fermented vegetables) are presented in Table 2. The vitexin-fermented vegetables had a significantly higher total phenolic content and significantly higher levels of all parameters of antioxidant activity than the probiotic-fermented vegetables and the standard fermented vegetables. In addition, the three formulas of fermented vegetables showed significant differences in TAC and DPPH on days 0 and 15 during storage, with the exception of ORAC in the standard fermented vegetables and FRAP in the standard and vitexin-fermented vegetables.

### 3.4. The Microbial Richness and Microbiota Diversity of Standard Fermented Vegetables, Probiotic-Fermented Vegetables, and Vitexin-Fermented Vegetables

**Figure 3:**
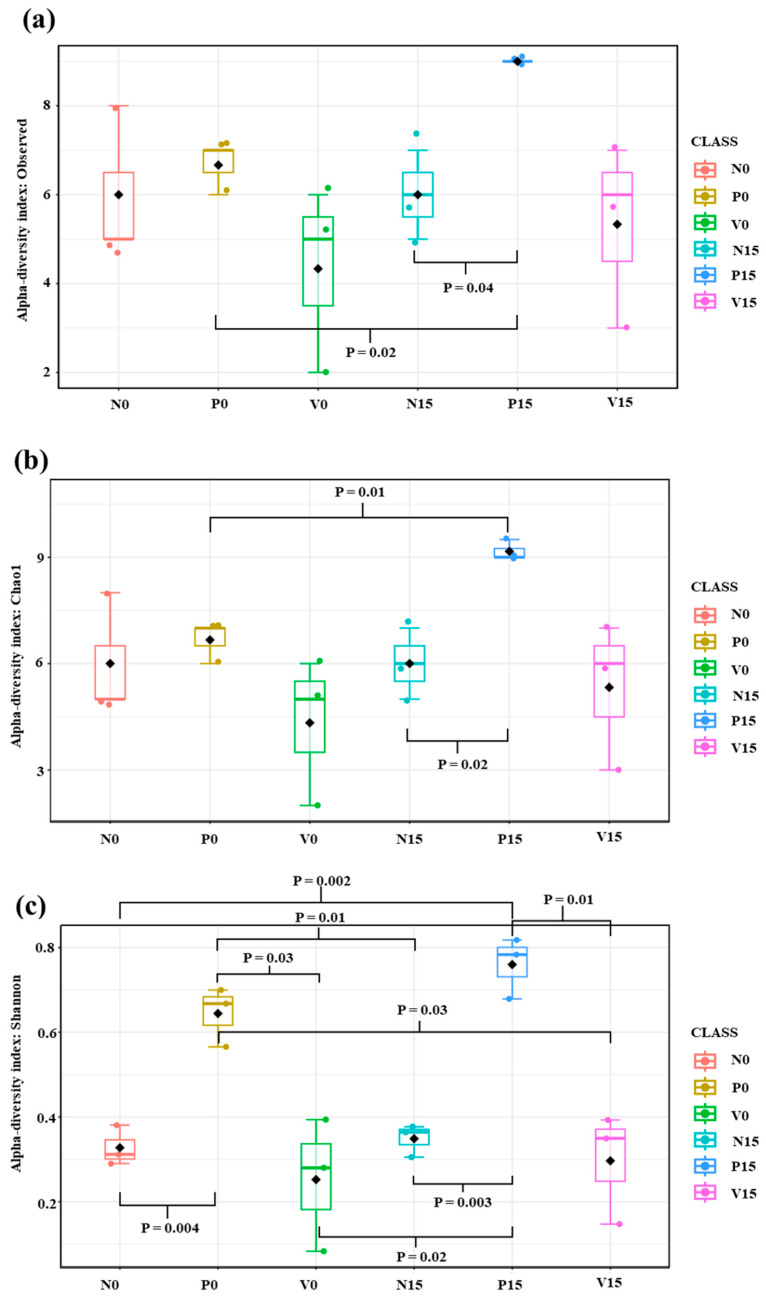
Alpha diversity among different fermented vegetable formulas (standard fermented vegetables, N group; probiotic-fermented vegetables, P group; and vitexin-fermented vegetables, V group) as measured using the number of observed species (**a**), the Chao1 index (**b**), and the Shannon index (**c**).

The standard fermented vegetables, probiotic-fermented vegetables, and vitexin-fermented vegetables were examined on days 0 and 15. After quality filtering, 100,797 high-quality sequence reads and 48 OTUs were obtained. Alpha diversity analysis refers to the richness of the microbiome; we analyzed the number of observed species (the number of OTUs), the Chao1 index (the richness), and the Shannon index (the richness and evenness distribution) (Figure 3). The Chao1 index and Shannon index showed the same trend as the number of observed species. We found significant differences in richness between the three formulas of fermented vegetables, including the number of observed species (N15 vs. P15, *p* value = 0.03; P0 vs. P15; *p* value = 0.03), Chao1 index (*p* value = 0.03; N15 vs. P15, P0 vs. P15), and Shannon index (*p* value = 0.0001; N0 vs. P0, N15 vs. P15, P0 vs. V0, and P15 vs. V15).

**Figure 4:**
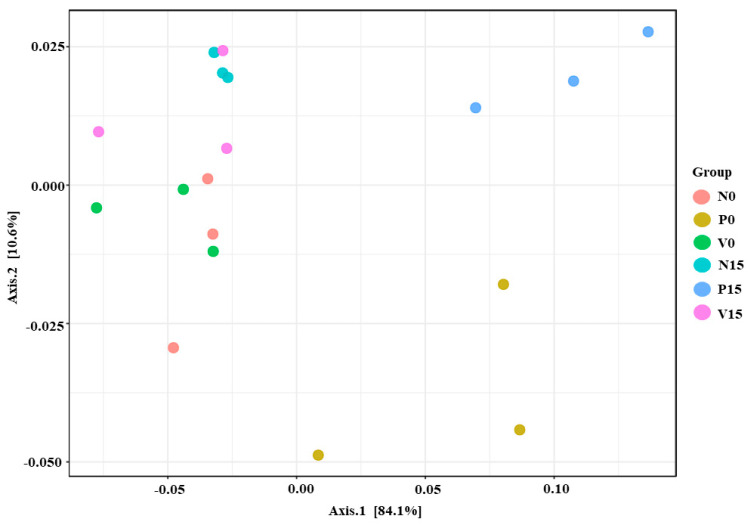
Principle coordinate analysis (PCoA) using Bray–Curtis distance analysis.

In this study, beta diversity was used to identify species differences between different environmental communities and was also used to evaluate the overall heterogeneity of the species or the environmental community. In addition, PCoA indicated significant differences in microbial composition between the three formulas of fermented vegetables (*p* value = 0.01, PERMANOVA) (Figure 4). These findings suggest that the observed differences in fermented vegetable formulas associated with storage time were likely related to bacterial richness and composition.

**Figure 5:**
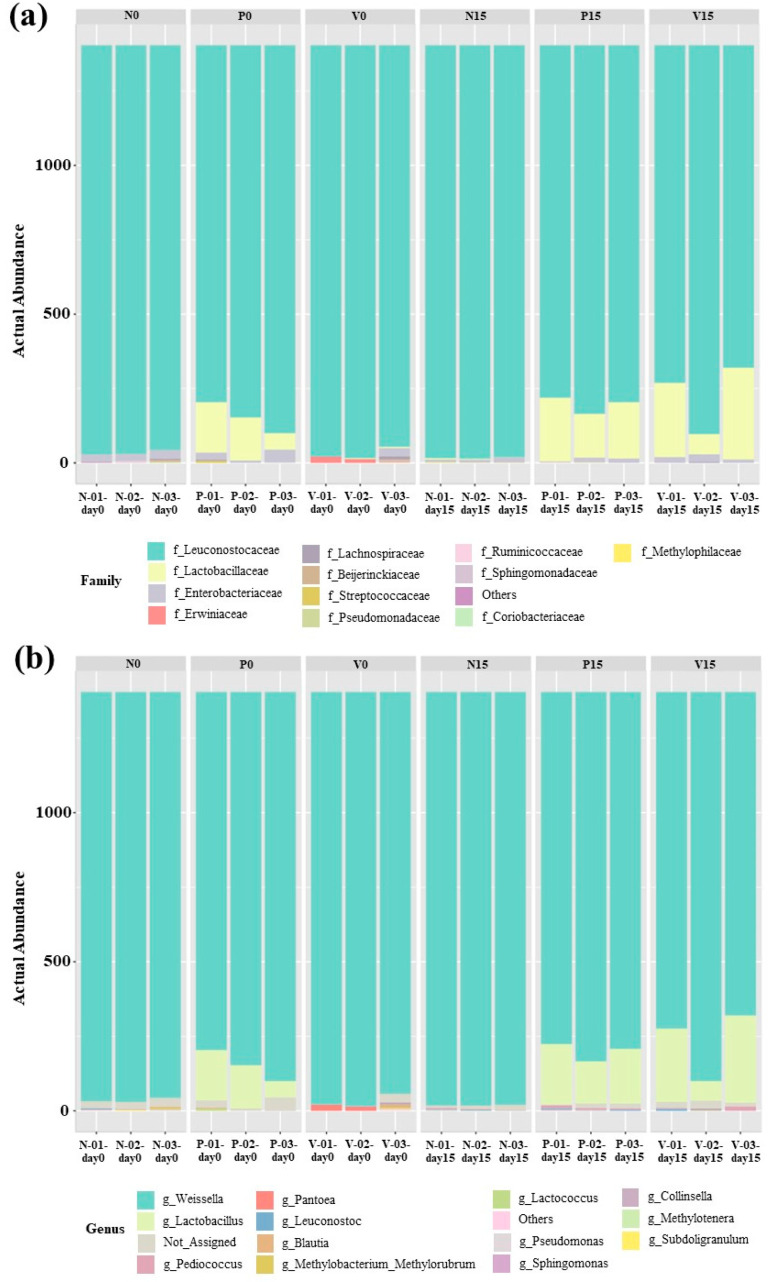
Abundances of bacterial operational taxonomic units (OTUs) at the family (**a**) and genus (**b**) levels. Samples are grouped according to the three fermented vegetable formulas (standard fermented vegetables, N group; probiotic-fermented vegetables, P group; vitexin-fermented vegetables, V group) at the storage times of 0 and 15 days.

The bacterial composition of the fermented foods differed according to the fermented food group. The relative abundance of OTUs in all fermented vegetable formulas at the family level is shown in Figure 5a. All of the analyzed sequences were classified into 12 bacterial families. *Leuconostocaceae* was the dominant family in all fermented vegetable formulas on days 0 and 15 of storage. In addition, a higher presence of *Lactobacillaceae* was observed in probiotic-fermented vegetables at days 0 and 15 and in vitexin-fermented vegetables at day 15. The family *Enterobacteriaceae* was observed in all fermented vegetable formulas during storage (days 0 and 15). Figure 5b shows the relative abundance of OTUs in all fermented vegetable formulas at the genus level (13 bacterial genera). *Weissella* spp. were predominant in all fermented vegetable formulas during the storage period. In the standard fermented vegetables, *Pediococcus* spp., *Leuconostoc* spp., and *Lactococcus* spp. were detected during storage. In the probiotic-fermented vegetables, the presence of *Lactobacillus* spp. together with *Pediococcus* spp. and *Leuconostoc* spp. was detected during storage. For the vitexin-fermented vegetables, *Pediococcus* spp. and *Leuconostoc* spp. were observed during storage, and *Lactobacillus* was the predominant bacterial genus at day 15 of storage.

**Figure 6:**
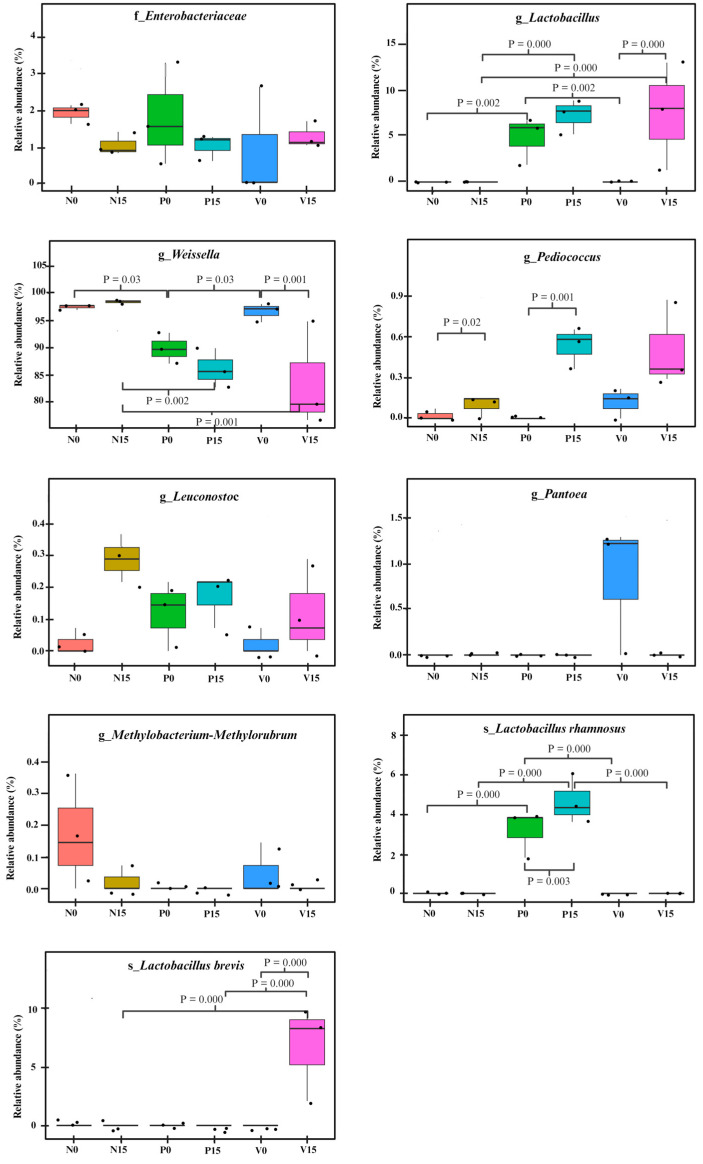
Comparison of the relative abundances of bacterial operational taxonomic units (OTUs) in the three fermented vegetable formulas (standard fermented vegetables, N group; probiotic-fermented vegetables, P group; vitexin-fermented vegetables, V group) at days 0 and 15 of storage. *p* values for significant differences were indicated.

We further analyzed the relative abundance of the OTUs between the three fermented vegetable formulas at different storage times. There were no statistically significant differences between members of the *Enterobacteriaceae* family or the genera *Pantora*, *Methylobacterium*-*Methylorubrum*, and *Leuconostoc*, when comparing the six fermented vegetable groups (No, N15, PO, P15, Vo, and V15), as shown in Figure 6. However, significant differences were observed in the genera *Pediococcus*, *Weissella*, and *Lactobacillus*, and the species *Lactobacillus rhamnosus* and *Lactobacillus brevis* (all *p* values < 0.05).

### 3.5. The Relationship between Bacterial OTUs and Antioxidant Activity

**Figure 7:**
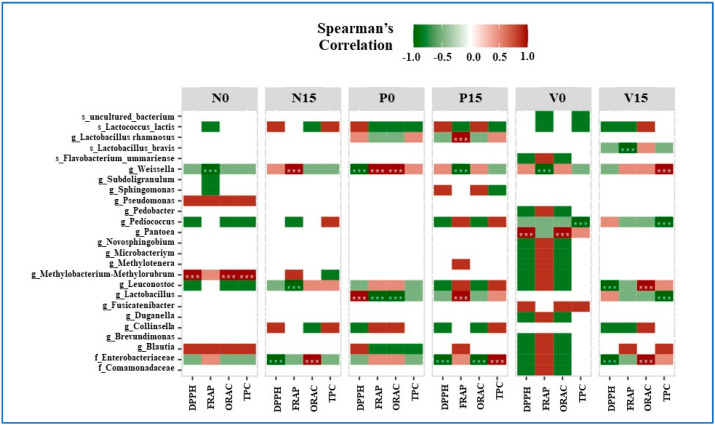
Spearman’s correlation coefficients for bacteria at the amplicon sequencing variant (ASV) level and for three parameters of antioxidant activity and the total phenolic content (DPPH, FRAP, ORAC, and TPC). Significance levels are indicated by asterisks (*** for *p* value < 0.05). Samples are grouped according to the three fermented vegetable formulas (standard fermented vegetables, N group; probiotic-fermented vegetables, P group; vitexin-fermented vegetables, V group) at days 0 and 15 of storage.

The relationship between bacteria, antioxidant activity, and total phenolic content (DPPH, FRAP, ORAC, and TPC) is shown in Figure 7. For the Spearman’s correlation coefficients, the positive and negative correlations are indicated in red and green, respectively. In the standard fermented vegetables, *Weissella* spp. were significantly negatively correlated with FRAP at day 0 and significantly positively correlated with FRAP at day 15. Methylobacterium-Methylorubrum spp. were significantly positively correlated with DPPH, ORAC, and TPC at day 0. *Leuconostoc* spp. were significantly negatively correlated with FRAP at day 15. Moreover, one amplicon sequencing variant belonging to the family Enterobacteriaceae was significantly negatively correlated with DPPH and significantly positively correlated with ORAC at day 15.

At day 0, *Lactobacillus* spp. were significantly positively correlated with DPPH but significantly negatively correlated with FRAP and ORAC in fermented vegetables containing probiotics. Furthermore, *Lactobacillus* spp. and *L. rhamnosus* had a strong positive correlation with FRAP at day 15. *Weissella* spp. showed a significant positive correlation with FRAP and ORAC on day 0, but a significant negative correlation with DPPH on day 0 and FRAP on day 15. At day 15, the bacterial family *Enterobacteriaceae* was significantly negatively correlated with DPPH and ORAC, and significantly positively correlated with TPC.

For vitexin-fermented vegetables, at day 15, *Lactobacillus* spp. showed a significant negative correlation with TPC, while *L. brevis* showed a significant negative correlation with FRAP. At day 0, there was a strong positive correlation between Pentoea spp. and both DPPH and ORAC. At days 0 and 15, there was a strong negative correlation between TPC and *Pediococcus* species. At day 0, *Weissella* spp. showed a substantial negative correlation with FRAP, and at day 15, a significant positive correlation with TPC. At day 15, *Leuconostoc* spp. showed a significant positive relationship with ORAC and a significant negative correlation with DPPH. Furthermore, by day 15, *Enterobacteriaceae* showed a strong positive association with ORAC and a strong negative association with DPPH.

**Figure 8:**
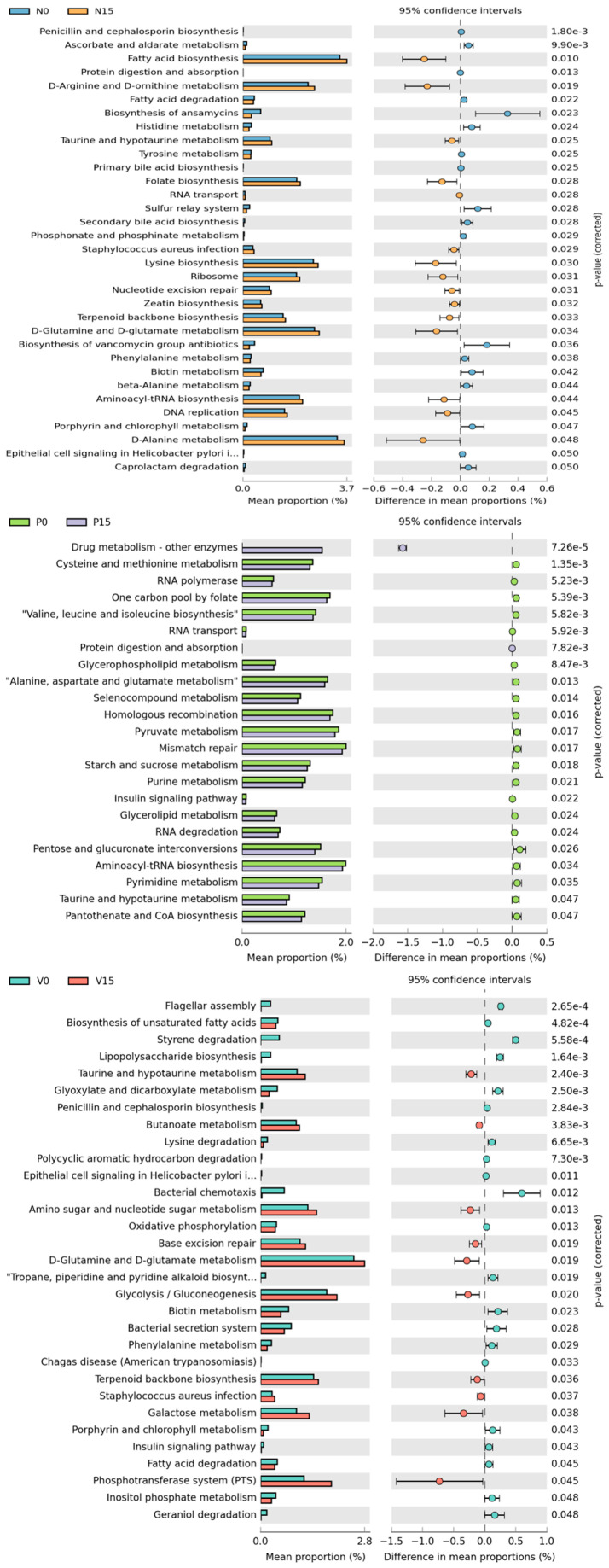
Predicted differential KEGG pathways in between day 0 and day 15 of three formulas of fermented vegetables. The extended error bar plots of significantly differential KEGG pathways were predicted using PICRUSt2 Version 2.5.2 analysis. Bar plots on the left side display the mean proportion of each KEGG pathway. Dot plots on the right show the differences in mean proportions between the two indicated groups using the p value. Only *p* values < 0.05 (Welch’s test) are shown.

Furthermore, metabolic pathway based on the core microbiome in the fermented vegetable samples was presented to predict different metabolisms related to functional analysis. The levels of various metabolic pathways among the different types of fermented vegetables were compared at day 0 with day 15 (Figure 8). For standard fermented vegetables, there were 33 significant metabolic pathways between storage times, including the biosynthesis of fatty acid, folate, lysine, secondary bile acid, and various metabolisms (D-arginine and D-ornithin, A-alanine, and histidine). Among 23 metabolic pathways found in probiotic-fermented vegetables, five of them were related to drug metabolism, cysteine and methionine metabolism, RNA polymerase, one carbon pool by folate and valine, leucine, and isoleucine biosynthesis. For vitexin-fermented vegetables, a varieties of metabolic pathways were observed with significant increases at day 15, including taurine and hypotaurine metabolism, amino sugar and nucleotide sugar metabolism, D-glutamine and D-glutamate metabolism, and glycolysis/gluconeogenesis.

## 4. Discussion

Fermented vegetables are popular in many countries, including Thailand. However, the use of different raw ingredients, preparation methods, and processing technologies results in considerable differences in the fermentation products. We evaluated the pH changes in the fermented vegetable formulas as this is an important physicochemical indicator of the anaerobic fermentation process. This process involves the microbial-driven degradation of carbohydrates resulting in organic acid and other chemical compounds [29]. In this study, the three fermented vegetables groups showed different pH values on day 0. These results were in agreement with those of a study on the effect of *L. rhamnosus* on pH changes in fermented plant-based raw materials [30], types of raw materials that could attribute to different pH levels in each sample. Vitexin-fermented vegetables had maltodextrin and gum arabic as carriers for the vitexin extract. These ingredients could result in a slight increase in the pH due to its basic property, whereas the probiotic-added fermented vegetables had *L. rhamnosus* GG, which has ability to slightly decrease the pH of the sample [30]. In fact, the pH values on day 0 of the probiotic-added fermented vegetables were measured at around 3–4 h after *L. rhamnosus* GG were added to the samples. Slightly different pH values (0.3) from the standard fermented vegetables (pH 4.2) could be observed, which were similar to those in previous studies [30,31]. In addition, decreases in pH from day 0 to day 15 in all of the fermented vegetable formulas were observed, similar to previous studies [30,31,32]. Probiotic-fermented vegetables containing *L. rhamnosus* underwent a process of heterofermentation to produce lactic acid and other organic acids, resulting in low pH levels throughout the storage period [32]. In another report, the pH of Baechu kimchi declined rapidly during the first week of fermentation and slowly thereafter [33]. Another study observed that kimchi stored at 4 °C on day 0 exhibited a pH of 5.8 that decreased gradually to 5.52 at week 2 and 4.9 at week 5, with the authors’ suggestion that storage at 4 °C promoted a longer and slower fermentation process [34].

In our study, there was a significant difference in the viable cell count of LAB in the probiotic-fermented vegetables on days 12 and 15 compared with that on day 0, whereas there was no significant difference in the viable cell count of *L. rhamnosus* over the storage period. These results follow the criteria established by the FAO/WHO [35], which state that products must maintain at least 106 to 107 CFU/mL of microorganisms during their shelf-life to be considered probiotic. In addition, these results were comparable to previous reports on an average LAB viable count of 8.01 ± 0.92 log CFU/g for kimchi (range 5.0–9.7) [36] and viable counts ranging from non-detectable to 8.7 CFU/g in 75 types of homemade fermented vegetables [37].

It has been reported that the effects of the fermentation process on plant-based foods results in many health benefits, including antioxidant activity due to the increase in the total phenolic compounds, and the magnitude of the effect depended on the species of microorganism [38]. In this study, vitexin-fermented vegetables showed the highest level of total phenolic compounds and antioxidant activity, as determined using the DPPH, ORAC, and FRAP methods. Vitexin is a polyphenol belonging to the class of flavones, and increases in functional ability may involve a metabolic change from large-molecule polyphenols, such as proanthocyanidins and flavonoid glycosides, to small-molecule polyphenols, such as aglycone and ellagic acid. Many beneficial bacteria in the fermentation process can metabolize polyphenol into small-molecule polyphenols, which exhibit stronger biological activity, including antioxidant, anti-inflammatory, and antimicrobial activities [15]. The total phenolic compounds and the antioxidant activities of probiotic-fermented vegetables were higher on day 15 than those at day 0, which was in accordance with other research, with the proposed mechanism relating to the microorganisms releasing enzymatically polymerized phenolic compounds that may be converted to simple phenolic compounds through hydrolysis. In addition, LAB exert their own antioxidative activities, increase the synthesis of antioxidant biomolecules and the expressions of antioxidant enzymes (SOD, CAT), and exhibit metal-chelating activities [38,39]. Consistent with a previous study, the antioxidant capacity (%) of *L. rhamnosus* GG-supplemented fermented vegetable juice during refrigerated storage was high at 84.62–90.21% [13].

To demonstrate the role of microorganisms in fermented vegetables, it is essential to identify the entire bacterial community and assess diversity and functional associations. The results of 16S rDNA-based PCR indicated significant alpha diversity between all three groups of fermented vegetables. *Weissella* spp., belonging to the *Leuconostocaceae* family, were dominant in all fermented vegetable formulas throughout storage. *Weissella* spp. have previously been detected in Korean temple fermented vegetables because of their presence in vegetables of the cabbage family (i.e., cabbage, cauliflower, and radish), which are the main ingredients of fermented vegetables [39,40]. The genus *Lactobacillus* was detected in probiotic-fermented vegetables at days 0 and 15, especially *L. rhamnosus* GG. The abundance of *L. rhamnosus* increased at day 15, possibly related to the decreased abundance of *Weissella* spp., which could promote the growth of *L. rhamnosus* by synthesizing extracellular polysaccharides. Moreover, EPSs can also resist pathogenic bacteria, including *Escherichia coli*, *Staphylococcus aureus*, *Streptococcus enterica*, *Listeria monocytogenes*, and *Bacillus cereus* [41]. The genus Lactobacillus was also detected in vitexin-fermented vegetables at day 15, especially *L. brevis*. *L. brevis* is also detected in the majority of Korean commercial fermented vegetables [42]. In this study, *L. brevis* was detected only in vitexin-fermented vegetables because only this formula had a suitable pH (pH 4.50 to 4.00), as the optimal pH value for the growth of this microorganism is within the range of 4.0 to 6.0 [43].

*Pedicoccus* spp. and *Leuconostoc* spp. were found to increase in abundance in all fermented vegetable formulas, especially in probiotic-fermented vegetables and vitexin-fermented vegetables, at day 15 of storage. *Pediococcus* spp. are present during the vegetable fermentation process, together with the genera *Lactobacillus* and *Leuconostoc* [44]. In addition, Pediococcus spp. have been reported in fermented vegetables in culture-dependent studies [41], indicating that the presence of the genus *Pediococcus* may be the result of supplementation with food additives such as vitexin. Regarding *Leuconostoc* spp., consistent with the results of Maoloni et al. [45], these species were present in fermented vegetables at the end of the fermentation process. Additionally, the metabolism of Leuconostoc spp. during fermentation can modify fructose to mannitol, resulting in a refreshing test of the vegetable fermentation process [46]. The genus *Pantoea* in the Erwiniaceae family was predominant only in vitexin-fermented vegetables at day 0 of storage and was assumed to originate from the raw ingredients. It has been reported that *Pantoea* spp. do not usually increase or decrease during fermentation [46], whereas *Methylobacterium*-*Methylorubrum* spp. tend to decrease in standard fermented vegetables and vitexin-fermented vegetables at day 15 [47] but are not detected in probiotic-fermented vegetables. Members of the Enterobacteriaceae family were detected in all fermented vegetable formulas at all storage times, but in low abundance. This is a large family of bacteria that comprises some pathogenic species, such as *Salmonella* spp., *Shigella* spp., and *E. coli*. Members of this family are often used as an indicator of hygiene in food production processes. As previously reported in vegetable-fermented foods, the abundance of LAB increases during fermentation, but then decreases because of the increased acidity caused by these bacteria [45,48].

The relationship between microorganisms and the antioxidant activities (DPPH, FRAP, ORAC, and TPC) of different fermented vegetable formulas at days 0 and 15 of storage was assessed using Spearman’s correlation analysis. *Weissella* spp. were dominant in all fermented vegetables at all storage times, and this correlated with antioxidant activity. This finding was consistent with that of a previous study that showed that some species of *Weissella* have antioxidant properties. *Weissella cibaria* isolated from Korean fermented vegetables can produce EPSs, which consist of phenols, flavonoids, glucoses, and galactoses, and has antioxidant properties that inhibit the production of free radicals such as reactive oxygen species [49]. The probiotic-fermented vegetables were detected to have higher abundances of the genus *Lactobacillus* and the species *L. rhamnosus* at day 15 than at day 0 of storage, which showed a significant and positive correlation with FRAP. Dzandu et al., previously reported the high antioxidant capacity of *L. rhamnosus*-supplemented fermented vegetable juice [13]. Similar to a previous study, four cereals (buckwheat, wheat germ, barley, and rye) fermented with *L. rhamnosus* showed a significant amount of total phenolics and high antioxidant activity (as determined using the DPPH and FRAP methods) [50]. The vitexin-fermented vegetables had a higher abundance of L. brevis at day 15 than at day 0 of storage, and this was significantly negatively associated with TPC and FRAP in contrast to a previous study that showed that *L. brevis* isolated from fermented vegetables has high antioxidant activity [51]. The findings of our study and previous reports are inconsistent with regard to *Pediococcus* spp. and antioxidant activity. The species *Pediococcus pentosaceus*, isolated from fermented foods, has high antioxidant activity (DPPH free radicals) and thereby inhibits lipid peroxidation [52]. Niknezhad et al. showed that *Pantoea* spp. produce novel EPSs, which act against free radicals and show good antioxidant activity [53]. Additionally, *Leuconostoc* spp., which were detected at day 15 of storage, showed a significant positive correlation with ORAC. A previous study showed that *Leuconostoc mesenteroides* exhibits antioxidant activity by producing EPSs [54]. *Methylobacterium*-*Methylorubrum* spp. were detected in the standard fermented vegetables at day 0 and showed a significant positive correlation with DPPH, ORAC, and TPC. This was consistent with the well-established antioxidant properties of Methylobacterium spp., which produces ergothioneine [55].

In addition to bacteria significantly associated with antioxidant activity, other microorganisms also contribute to antioxidant activity but are present in lower abundance. However, some microorganisms show an ambiguous relationship with TPC and the three parameters of antioxidant activity. This may reflect synergistic interactions between antioxidants in fermented foods that contain various microorganisms, meaning that the antioxidant activity is not only dependent on the antioxidant concentration, but also on the structure and interactions of the antioxidants. Moreover, TPC does not include all antioxidants, such as carotenoids and tocopherols [50,56]. This may explain why some microorganisms correlated with TPC but not with antioxidant activity. An active interaction of bacteria function complexity based on KEGG pathways, including nutrients biosynthesis, lipid metabolism, amino acid metabolism, carbohydrate metabolisms and secondary bile acid was found in this study. Similarly, a previous study showed three predicted metabolic pathways, including membrane transport, replication and repair, and translation, as the most abundant in all species of the fermented vegetable samples from China [57]. This information provides a comprehensive insight of the potential of bacteria in fermented products, which is of great significance of the bacterial diversity in the development of healthy food and intervention studies.

There were several limitations in this study. Total measurements of pH and microbial counts for all formulas with serial storage times should be performed in further studies. Increasing the sample size of each fermented vegetable group can provide findings for the effective production of healthy fermented vegetable foods. In addition, conducting a metabolome analysis for all groups of fermented vegetables will provide the core microorganisms related to the changes in important metabolites during fermentation and the main mechanisms underlying health benefits.

## 5. Conclusions

In this study, the predominant microorganisms in fermented foods at the family, genus, and species taxonomic levels were analyzed, and the correlations between microbial diversity and antioxidant activity were investigated. Significant differences in the bacterial communities and the antioxidant activities of the three formulas of fermented vegetables during storage were detected. The findings from the present study also confirm that the raw ingredients and food additives used to supplement fermented foods result in different bacterial communities and antioxidant activities. In particular, supplementation with *L. rhamnosus* GG and vitexin, rich probiotics, can increase the abundance of LAB, suppress pathogenic bacteria, and enhance antioxidant activity. However, further studies are needed to investigate the relationship between microorganisms and antioxidant activity in fermented foods, specifically by analyzing the mechanisms, pathways, or microbial metabolite profiles of fermented foods during storage that may affect antioxidant activity.

## Figures and Tables

**Table 1:** Viable counts (log CFU/g) of *Lacticaseibacillus rhamnosus* GG and lactic acid bacteria in fermented vegetables supplemented with probiotics (n = 3).

Formula	Storage Time (Days)	*Lacticaseibacillus rhamnosus* GG	Lactic Acid Bacteria
Fermented vegetables supplemented with probiotics	0	7.71 ± 0.24 ^a^	8.53 ± 0.19 ^a^
	3	7.68 ± 0.12 ^a^	8.42 ± 0.09 ^a^
	6	7.60 ± 0.10 ^a^	8.26 ± 0.19 ^a^
	9	7.60 ± 0.17 ^a^	8.21 ± 0.23 ^a^
	12	7.66 ± 0.04 ^a^	8.08 ± 0.08 ^ab^
	15	7.48 ± 0.40 ^a^	7.61 ± 0.30 ^b^

Values are expressed as the mean ± standard deviation (SD). Different superscript letters indicate a significant difference (*p* < 0.05).

**Table 2:** Antioxidant activity of standard fermented vegetables (N), probiotic-fermented vegetables (P), and vitexin-fermented vegetables (V).

Antioxidant Activity	Formulas	Storage Time	
		Day 0	Day 15
TPC (mg GAE/g sample)	N	1855.00 ± 54.95 ^a^	2161.44 ± 51.84 ^a,^*
	P	2038.33 ± 20.21 ^b^	2490.10 ± 78.93 ^b,^*
	V	3990.45 ± 94.61 ^c^	4925.63 ± 40.53 ^c,^*
DPPH (µmol TE/g sample)	N	10,257.79 ± 754.45 ^a^	12,494.70 ± 529.56 ^a,^*
	P	10,344.70 ± 465.36 ^a^	13,350.78 ± 878.67 ^a,^*
	V	16,232.39 ± 532.06 ^b^	19,645.62 ± 163.91 ^b,^*
ORAC (µmol TE/g sample)	N	32,252.04 ± 1771.88 ^a^	34,429.51 ± 1838.23 ^a^
	P	69,616.58 ± 1131.59 ^b^	78,285.27 ± 1467.08 ^b,^*
	V	88,466.93 ± 1607.07 ^c^	128,401.30 ± 2492.60 ^c,^*
FRAP (µmol TE/g sample)	N	10,167.19 ± 227.09 ^a^	11,380.52 ± 877.75 ^a^
	P	10,559.74 ± 845.03 ^a^	13,005.10 ± 732.32 ^a,^*
	V	35,351.76 ± 1867.80 ^b^	40,100.52 ± 1435.23 ^b^

Values are expressed as the mean ± standard deviation (SD). Different superscript letters represent significant differences between groups on the same day (*p* < 0.05). The asterisks (*) indicate significant differences within a group on a different day (*p* < 0.05).

## Data Availability

The original contributions presented in the study are included in the article, further inquiries can be directed to the corresponding author.
